# Herd Behaviour, Fundamental, and Macroeconomic Variables – The Driving Forces of Stock Returns: A Panel-Based Pooled Mean Group Approach

**DOI:** 10.3389/fpsyg.2022.758364

**Published:** 2022-03-21

**Authors:** Shaista Jabeen, Sayyid Salman Rizavi, Muhammad Farhan

**Affiliations:** ^1^Department of Management Sciences, Lahore College for Women University, Lahore, Pakistan; ^2^Hailey College of Commerce, University of the Punjab, Lahore, Pakistan; ^3^Department of Management Sciences, National University of Modern Languages, Islamabad, Pakistan

**Keywords:** herd behaviour, stock returns, panel ARDL, Pakistan stock exchange, CSAD

## Abstract

The existing research aims to seek the herding effects on stock returns at the industry level in Pakistan Stock Exchange (PSX). Moreover, the relationship between stock returns and herding has been studied by taking some macroeconomic (exchange rate, interest rate, and inflation rate) and fundamental (return on equity and earnings per share) control variables. Herding is actually imitating other’s behaviour. This phenomenon indicates a situation where the investors follow the crowed and ignores their personal information, despite knowing the correctness of their information. Herd behaviour may drive from fundamental factors leading to efficient markets. However, it may not be associated with fundamental information causing unstable prices. The stock price data of PSX listed companies from 1999 to 2017 have been the point of focus. The underlying herding measure was the cross-sectional absolute deviation (CSAD), proposed by Chang et al. (2000). The significant analysis technique facilitating the current research is pooled mean group (PMG)/panel autoregressive distributed lag (ARDL) approach. Findings revealed that some sectors are evident for positive effect of herding on stock returns, whereas some others are witnessed for its negative effects on stock returns, in both long run and short run. As far as the control variables are concerned, they demonstrated both significant and insignificant effects on stock returns in different sectors of PSX. The study has important implications for policymakers.

## Introduction

Investors’ rationality is the foundation of behavioural finance, which establishes a vital distinction against the assumptions of efficient market hypothesis (EMH). Further, it has been argued that the markets having only rational approach participants are impracticable to develop (Shleifer, 2000). EMH implies that investors are inclined toward active investment behaviour and then following the passive behaviour. However, investors follow or imitate the decision of other investors based on their irrational attitude. Behavioural imitation of investors is termed as herding, which leads the investors to a situation where they set aside their personal information to follow the crowd, even the realisation of their accurate personal information. Investors do it consciously or unconsciously or it can be a deliberate decision or an intellectual bias (Van Campenhout and Verhestraeten, 2010).

The financial models such as capital asset pricing model (CAPM) and arbitrage pricing theory (APT) describe the investors’ support toward EMH in terms of correct evaluation of financial securities by observing rational outlooks. In addition, these two models also assist in the best explanation of stock returns. These models further undertook the different economic variables that cause stock returns (Bessler and Opfer, 2004). The point of concentration of these models is on exploring factors that affect stock returns; i.e., the market return is solely responsible for influencing the stock returns, or some other factors are involved.

According to Sarwar et al. (2019) the stock markets are believed to be very much subjective toward macroeconomic variables. Considering the stock market of Pakistan, Securities and Exchange Commission of Pakistan (SECP) has the responsibility to regulate all the capital markets of Pakistan (Pakistan Economic Survey, 2012–2013). SECP monitors self-regulatory organisations (SRO), growth of capital markets, and intermediaries operative in capital markets. SRO consists of Pakistan Mercantile Exchange Limited (PMEX), Central Depository Company of Pakistan Limited (CDC), National Clearing Company of Pakistan Limited (NCCPL), and Pakistan Stock Exchange (PSX). The intermediaries and agents include ballotters, share registrars, underwriters, advisors, managers, and brokers. Previously, there were three stock exchanges of Pakistan, i.e., Islamabad Stock Exchange, Karachi Stock Exchange, and Lahore Stock Exchange. All these stock exchanges were merged on 11 January, 2016 and were given the name of Pakistan Stock Exchange. It is a sole platform to investors for performing the trading activities in Pakistan. PSX is considered to be one of the best performing stock exchange markets of Asia, and it secured 3rd and 5th positions worldwide in 2014 and 2016, respectively.

The researchers are aiming to examine the impact of herding on stock returns along with associated macroeconomic and fundamental control variables. As per the limited knowledge of researchers, herding has been thoroughly investigated in Pakistan’s context. Scholars have presented the mixed evidence of herding in the KSE-100 index or in the overall Pakistan stock market. Some have found its evidence (Malik and Elahi, 2014; Zafar and Hassan, 2016; Qasim et al., 2019), some have reported partial evidence (Shah et al., 2017; Khan and Rizwan, 2018; Yousaf et al., 2018; Jabeen and Rizavi, 2019), and some have highlighted its absence (Javed et al., 2013; Javaira and Hassan, 2015; Kiran et al., 2020).

Moreover, in the context of Pakistan, the researchers have found the impact of macroeconomic variables and fundamental variables on stock returns (Atiq et al., 2010; Butt et al., 2010; Mahmood et al., 2015; Afshan et al., 2018; Khan et al., 2018). The international researchers have also explored such relationship (Olugbenga and Atanda, 2014; Abdulmannan and Faturohman, 2015; Mitra, 2017; Ahmed, 2018; Ndlovu et al., 2018; Saragih, 2018). Some international studies have investigated the impact of herding on stock returns (Demirer et al., 2015; Chen and Demirer, 2018; Hwang et al., 2018; Rompotis, 2018). Also, researchers have found a study in the local context in which the impact of macroeconomic variables on herding has been investigated, followed by insignificant results (Javaira and Hassan, 2015).

In such perspective, the current research is novel in the sense it has examined the impact of herd behaviour on stock returns with the macroeconomic (exchange rate, inflation rate, and interest rate) and fundamental variables (earnings per share and return on assets). Another contribution of this manuscript is the inclusion of 34 sectors of PSX instead of just targeting the KSE-100 index. The study has also focused on the extended time span, i.e., 1999–2017, whereas the previous studies have been conducted for some specific time (2004 onward). Besides, the significant contribution of the current research is the application of panel autoregressive distributed lag (ARDL) and pooled mean group (PMG) technique, which has provided novelty to this study as to date, and as per the limited knowledge of researchers, PMG technique has not been applied in herding context especially in Pakistan. Indeed, the non-stationarity panel has guided the adoption of such technique (Sare et al., 2018; Yao and Hamori, 2018).

## Literature Review

Herd behaviour in the financial markets has been discussed in detail in the already available literature. The 1st empirical study to examine market-wise herd behaviour was carried out by Christie and Huang (1995). The study found no proof of herd behaviour in the market of the United States. According to the authors, the investors use their personal information to make decisions while acting rationally; however, during extreme market conditions, they follow the crowd. The authors found an indication of herd behaviour during the panic market conditions in Taiwan and South Korea. Afterward, a study conducted by Chang et al. (2000) also found assertive herd behaviour in Taiwan and South Korea. However, they found no evidence in Hong Kong and United States stock market and minimal effect in the Japanese market. Their conclusion about the presence of herding during extreme market conditions was based on a non-linearity test.

Chiang and Zheng (2010) investigated herding effects by utilising a modified version of CSAD in 18 global markets from 1998 to 2009. According to their research, herd behaviour was found in Asian markets and developed markets except for the United States. On the other hand, herd behaviour was found in Latin markets and the United States market during crisis periods. Herd behaviour was also investigated by Doğukanlı and Ergün (2011) in Borsa Istanbul, but no evidence of herding was found. Likewise, herd behaviour was not observed by Al-Shboul (2012) in the Jordanian market, before and after the financial crisis of 2008, using the cross section standard deviation (CSSD) methodology. Herding has been reported by the CSAD method only in the crisis time. Ahsan and Sarkar (2013) used the same method to investigate the herd behaviour in the Dhaka Stock Exchange, but no herding signs were found. The authors explained that investor’s decisions are based on the information available to them instead of following the market trend.

Furthermore, Messis and Zapranis (2014) observed herding and its impact on the market volatility in the Athens market by utilising Hwang and Salmon (2004) measure. Herding was investigated in the Spanish stock market (Ahmed et al., 2015) after, during, and before the global financial crisis of 2008 by utilising the above-discussed measures. Herd behaviour was also highlighted in China’s up and down stock market through CSAD method (Chong et al., 2016). Herding was attributed to risk factors; short-term investment aptitude of the investors, and analyst recommendation, but it was not related to the firm’s size. During the significant crisis in France, herd behaviour was examined by Litimi (2017) using the CSAD model in the stock market at the sectoral level. Mertzanis and Allam (2018) utilised daily and monthly data to find the herd behaviour in the Egyptian stock market during bear and bull market conditions, but no herd behaviour was found. They inferred that herd behaviour is a short-living aspect. Bui et al. (2018) utilised CSAD and CSSD models and found herd behaviour in the frontier stock market, especially in the stock market of Vietnam at both market and industry levels (up/down market). Furthermore, the stock market of Vietnam was influenced by the United States stock market, but Hong Kong stock market was only affected by the market information.

Vo and Phan (2019a,b) found herding in the equity market of Vietnam. Herding was investigated by Can and Dizdarlar (2019) in Istanbul’s stock market from 2011 to 2017. They found herding at 1%, but at 5%, it prevailed only during upper extremes. Herd behaviour in the Balkan Stock Exchanges (Solvenia, Romania, Croatia, and Bulgaria) from 2000 to 2016 utilises CSAD model with a primary focus on the Eurozone crisis and global financial crisis (Economou, 2019). The herd behaviour was exhibited in the Balkan region as a whole as compared to the individual stock markets, with the Romanian stock market exhibited the most substantial effect among all other markets. Using the quantile regression and CSAD model, Shantha (2019) studied the evolutionary nature of herd behaviour from 2000 to 2008 in Colombo stock market. Herd behaviour was found to be of twisted nature. Stavroyiannis and Babalos (2019) observed negative herding in the Eurozone stock markets from 2000 to 2016 by utilising CAPM-based procedure. Using the CSAD measure, Ju (2019) explained the prevalence of herding in A and B share markets. The investors of B share herd on various styles such as growth and value stocks, whereas A share stock market herd on growth stocks.

Batmunkh et al. (2020) found herd behaviour in the Mongolian stock market from 1999 to 2019 during bullish and bearish market trends. Wanidwaranan and Padungsaksawasdi (2020) established the strong impact of return jumps on global equity markets. Kumar et al. (2020) investigated herd behaviour in major Asian commodity future markets. Heterogeneous herding effects were observed in these economies. Chauhan et al. (2020) followed aggregate herding signs in large-cap Indian stocks. Mand and Sifat (2021) found herd behaviour in Bursa Malaysia and explained that herding was regime-dependent and non-linear approach. Ukpong et al. (2021) did not observe herd behaviour in the overall United States stock market, but it was profound at the industry level.

### Factors Affecting Stock Returns

The relationship of stock returns with macroeconomic and fundamental variables is gaining importance over time. These factors can be external (political incidents, war, oil prices, inflation rates, exchange rates, interest rates, etc.) and also internal (management quality, financing, type of investment, etc.) (Butt et al., 2010; Chen and Chiang, 2016).

According to Waheed et al. (2018), different studies have also examined the relationship between stock returns and macroeconomic variables in developed and developing economies. The already available literature has explained the relationship of fundamental factors with the stock returns. Zulkarnaen et al. (2012) demonstrated that an institution’s ability to generate high returns boosts the stock returns. Suresh (2013) described that fundamental analysis is used to study the impact of macroeconomic factors and firm-specific factors on the stock returns. Mirfakhr-Al-Dini et al. (2011) examined a positive relationship between stock prices and earning per share. The authors also observed a negative relationship of stock prices with price-earnings (PE) ratio and dividend per share (DPS). The same association was explained by Emamgholipour et al. (2013). Glezakos et al. (2012) also described a significant positive relationship of book value per share and earnings per share (EPS) with the stock returns. Various other researchers presented the same results (Ikhatua, 2013; Menike and Prabath, 2014; Olugbenga and Atanda, 2014). Khan et al. (2012) found a positive impact of earnings yield and dividend yield on stock returns and a negative impact of market ratio on the stock returns. Shehzad and Ismail (2014) explained that book value per share and earning per share have a significant positive relationship with the stock returns. Vijitha and Nimalathasan (2014) found a significant positive association of asset value per share, PE ratio, return on equity (ROE), and EPS with the stock returns. The same results were also reported by Zeytinoğlu et al. (2012). Haque and Faruquee (2013) reported the same results in Dhaka Stock Exchange. Ahmed (2018) found significant positive impact of DPS and EPS on stock returns. Saragih (2018) reported that ROE and ROA have no impact on the stock returns.

In the existing literature, we can explain the relationship of stock returns with interest rate and inflation rate. Butt et al. (2010) found that an economy’s inflation factor negatively relates to the stock returns. Allahawiah and Al-Amro (2012) described that stock prices are mostly affected by the inflation rate. Khan et al. (2018) also reported a similar relationship. On the other hand, Ndlovu et al. (2018) found a positive relationship between stock returns and the inflation rate. Khan (2014) observed a positive association of stock returns in the KSE-100 index with the inflation rate. Khan (2014) and Khan et al. (2018) highlighted the negative relationship of stock returns of the KSE-100 index with the interest rate. Conversely, Kibria et al. (2014) found a positive relationship between stock returns and interest rate. Ndlovu et al. (2018) explained that stock returns are positively affected by the long run’s interest rate.

Stock return is affected by the exchange rate in different ways. For instance, Fang and Miller (2002) identified that currency depreciation has a significant relationship with stock returns. Prices of imported goods are increased by the devaluation of local currency, which ultimately reduces import-reliant institutions’ net income (Erdogan and Ozlale, 2005). Dornbusch and Fischer (1980) explained the positive relationship between stock returns and exchange rates through the goods market theory. On the other hand, portfolio balance theory highlights a negative relationship between these two variables (Branson, 1983). Khan (2014) studied a positive relationship between stock returns and exchange rate in KSE-100 index. The same association was identified by Sohail and Hussain (2009) in KSE but not in ISE and LSE. Bahmani-Oskooee and Saha (2016) and Khan et al. (2018) explained the positive relationship of the exchange rate with the stock returns; however, Menike (2006) examined the negative connection between these two variables. A long-run association between stock returns and exchange rate were identified by Türsoy (2017). The long-run negative linkage between stock returns and exchange rate was found in India, and a long-run positive association between these two variables was observed in Russia and Brazil.

### Herd Behaviour and Stock Returns

The existing literature has pointed out herding’s presence in the stock markets; however, the medium linked with the impact of herding on the security prices is required to be explored. To discover the influence of herd behaviour on security return is a topic of interest among academicians because of its role in profitable investments (Litimi et al., 2016; Vinh and Anh, 2016).

Herd behaviour is an appealing phenomenon explaining the volatility of asset returns and security prices (Dasgupta et al., 2011). It may have a positive or negative impact on the stock returns. Jeon and Moffett (2010) investigated the relationship between stock returns and herd behaviour in the emerging markets. The authors found a significant effect of herd behaviour on stock returns. Hsieh (2013) investigated the presence of individual and institutional herding in stock market of Taiwan. A novel approach to measure the tracking dynamics of herding for individual investors was presented by Merli and Roger (2013). According to the authors, herding was presented in the market and became consistent over time. Latief and Shah (2014) explained that herd behaviour of mutual funds has a significant relationship with the stock returns.

Herd behaviour plays a crucial role in defining the relationship between future and past returns (Demirer et al., 2015; Chen and Demirer, 2018). Hwang et al. (2018) examined the impact of herding on stock returns by utilising network theory. Chen and Demirer (2018) explained that herd behaviour influences return or profit generation. The author’s used state space, CSAD, and CSSD methods to conclude that herding generates significant profits and high returns. Furthermore, herding is considered as an important driver of returns and profitability. Herding can be considered as a root cause of low stock returns.

### Study Contributions

This study has been aimed to add in the already available literature of herding, and it also provides some novel insights regarding the herding behaviour in the Pakistani stock market. As far as Pakistan is concerned, limited studies are available which have focused herding in Pakistani stock market (Javed et al., 2013; Malik and Elahi, 2014; Javaira and Hassan, 2015; Zafar and Hassan, 2016; Shah et al., 2017; Khan and Rizwan, 2018). Majority of these studies have focused on KSE-100 index by utilising monthly and daily returns. This study has emphasised on overall trend of PSX along with all of its sectors. A comprehensive picture has been obtained by utilising daily stock returns regarding the long-term and short-term herding phenomenon. Furthermore, application of PMG method has also brought novelty to this research as this methodology has not been applied in Pakistan with respect to herding. Moreover, according to the limited knowledge of the authors, only one study has focused on the impact of herding on stock returns (Latief and Shah, 2014), and this study has only investigated mutual funds’ herding. This study also adds to the current body of knowledge by examining the impact of herding on stock market returns by utilising various fundamental and macroeconomic variables and by utilising the most appropriate methodology, i.e., panel ARDL/PMG.

## Research Methodology

### Data Description

This study examines herding’s effects on stock returns in PSX. It has also used macroeconomic and fundamental control variables in assessing the impact of herd behaviour on stock returns. Trading data of 528 listed companies, categorised in 34 sectors, have been gathered from 1999 to 2017, from PSX and business recorder’s official website. Yearly data of macroeconomic variables have been taken from the World Bank website, and the fundamental data have been collected from the annual reports of respective companies. For some financial sectors, data availability was ensured from 2004 to 2017. Following this, in these specific sectors, data symmetry has been maintained; i.e., the herding, stock returns, and control variables data were accordingly adjusted. It is to be noted here that the sectors having herding even in a single year were targeted for investigating the association, whereas the sectors where herding was not observed in any single year were excluded from the analysis.

### Measurement of Variables

Table 1 indicates the measurement of variables of the study.

**TABLE 1:** Measurement of variables.

Variable	Measurement
Exchange rate (ER)	Annual rupees per unit of United States Dollar
Interest rate (IR)	Annual lending rate
Inflation rate (INR)	Consumer price index
Earnings per share (EPS)	Net income/outstanding shares
Return on assets (ROA)	Net income/total assets
Stock returns (SR)	*Ln*(*P_*t*_/P_*t*–1_*)
Herd behaviour (HB)*	*Existence of herding* = *1/Absence* = *0 Non existence of herding = 0*

*Presence/absence of herding is illustrated by creating its dummies. The presence of herding implies “1” and “0” otherwise.

#### Model of Herd Behaviour

Cross-sectional absolute deviation is the underlying herding model. This measure focuses on the dispersion of single security returns and the weighted average market returns. It also takes into account the extreme market movements. Herding leads single security returns to group around the market returns, ultimately directing investors to set aside their information and follow the crowd. Besides, it also focuses on the non-linear association between stock return and market returns. The basic equation of this model (1), the equation to represent the non-linear connection (2), and the equation for explaining the bullish and bearish trends (3) are presented as follows:


(1) $$\mathrm{CSAD}=\frac{1}{N}\,\,\sum_{i=1}^{N}\left|R_{i,t}-R_{m,t}\right|$$

(2) $$C\,S\,A\,D=\alpha +\gamma_{1}\,\left|R_{m,t}\right|+\gamma_{2}\,R_{m,t}^{2}+\varepsilon$$

*R*_*m,t*_ = Market return.

*Y_2_* = If, significant and negative, infers herd behaviour.


(3) $$C\,S\,A\,D_{t}^{U\,P}=\alpha +\gamma_{1}^{U\,P}\,\left|R_{m,t}^{U\,P}\right|+\gamma_{2}^{U\,P}\,{\left(R_{m,t}^{U\,P}\right)}^{2}+\varepsilon_{t}\,\text{if}\,R_{m,t}^{U\,P}>0$$

(4) $$C\,S\,A\,D_{t}^{D\,O\,W\,N}=\alpha +\gamma_{1}^{D\,O\,W\,N}\,\left|R_{m,t}^{D\,O\,W\,N}\right|+\gamma_{2}^{D\,O\,W\,N}\,{\left(R_{m,t}^{D\,O\,W\,N}\right)}^{2}+\varepsilon_{t}\,\text{if}\,R_{m,t}^{D\,O\,W\,N}<0$$

*Y_2_* = If, significant and negative, infers herd behaviour.

$R_{m,t}^{U\,P}$ = Equally weighted portfolio returns during bullish phase.

$R_{m,t}^{D\,O\,W\,N}$ = Equally weighted portfolio returns during a bearish phase.

$C\,S\,A\,D_{t}^{U\,P}$ = CSAD at time *t* during rising market trends.

$C\,S\,A\,D_{t}^{D\,O\,W\,N}$ = CSAD at time *t* during falling market trends.

### Analysis Technique (Pooled Mean Group)

Panel autoregressive distributed lag proposed by Pesaran et al. (1999) was the underlying analysis technique to assess herding’s impact on stock returns. Non-stationarity panels lead to the application of such technique. It deals with mean group (MG) and PMG techniques (Okunade et al., 2018), which are appropriate for the panels having large cross-sections and time dimensions (Tan, 2009). PMG technique implies pooling of data and taking its averages on the assumptions that slope coefficients and error variances remain the same across all groups; however, intercepts may have different values. Also, this technique asserts that long-run regression coefficients continue to be the same but the error variances and short-run coefficients may vary (Tan, 2009; Okunade et al., 2018). PMG strictly deals with the homogeneity for long-run groups’ coefficients and also significant and negative error correction term (Yao and Hamori, 2018). Its results are independent of outliers and lag order selection (Umar and Nayan, 2019).

The following regression equations have been derived based on the measurement of the variables of study:


(5) $$S\,R_{i,t}=\beta_{0}+\beta_{1}\,E\,R_{i,t}+\beta_{2}\,I\,R_{i,t}+\beta_{3}\,I\,N\,R_{i,t}+\beta_{4}\,H\,B_{i,t}+\beta_{5}\,E\,P\,S_{i,t}+\in_{i,t}$$

(6) $$S\,R_{i,t}=\beta_{0}+\beta_{1}\,E\,R_{i,t}+\beta_{2}\,I\,R_{i,t}+\beta_{3}\,I\,N\,R_{i,t}+\beta_{4}\,H\,B_{i,t}+\beta_{5}\,R\,O\,A_{i,t}+\in_{i,t}$$

## Empirical Results

Descriptive statistics of various sectors of PSX have been summarised in Table 2. The summary contains all the variables of study, i.e., return on equity, earning per share, herd behaviour, inflation rates, interest rates, exchange rates, and stock returns. Numbers of observations, maximum values, minimum values, standard deviation, and mean for each sector and each variable have also been reported. For macroeconomic factors, i.e., inflation rates, interest rates, and exchange rates, across all sectors, the data for these variables are same across all sectors. It is reported once in the table showing the summary of descriptive statistics. The standard deviation and mean of Inflation Rate (INR) follow the range of 4–7, whereas Exchange Rate (ER) lies in the range of 79–19, and Interest Rate (IR) falls in the range of 2–11. The higher values of mean indicate a significant surge in market differences across sectoral returns. On the other hand, the high values of standard deviation indicate abnormal cross-sectional disparities in various sectors of the stock market because of shocks or unanticipated events. The standard deviation and mean values of herd behaviour also suggest a similar trend across all the sectors. Furthermore, the statistics about the fundamental factors, i.e., Return on Assets (ROA) and Earnings per Share (EPS), show that different sectors follow different directions based on their financial performance. Some corporations earn a high return on their assets, and some corporations pay significantly high earnings to investors because of favourable earnings.

**TABLE 2:** Descriptive statistics.

S#	Variables	Observations	Mean	Standard deviation	Minimum	Maximum
**(1) Automobile assembler**						
	SR	228	0.064	−*0*.*901*	0.289	2.001
	ER	228	79.579	19.677	51.9	110.433
	IR	228	11.106	2.289	7.25	16
	INR	228	7.762	4.52	2.53	20.29
	HB	228	0.052	0.223	0	1
	EPS	221	19.666	29.752	−*28*.*3*	165.41
	ROA	222	0.08	0.127	−*0*.*309*	0.539
**(2) Automobile parts and accessories**						
	SR	171	0.043	0.291	−*1*.*341*	1.169
	HB	171	0.21	0.408	0	1
	EPS	155	11.73	18.815	−*24*.*06*	95.16
	ROA	155	0.066	0.142	−*0*.*503*	0.385
**(3) Cable and electrical goods**						
	SR	133	0.024	0.296	−*0*.*691*	1.183
	HB	133	0.308	0.105	0	1
	EPS	113	11.569	51.637	–118.17	300.77
	ROA	111	–0.007	0.1	–0.326	0.185
**(4) Cement**						
	SR	382	0.025	0.238	–0.918	0.877
	HB	380	0.157	0.365	0	1
	EPS	334	2.826	8.572	–37.5	42.34
	ROA	329	0.036	0.105	–0.36	0.356
**(5) Close-end mutual funds**						
	SR	55	0.007	0.246	–0.674	0.871
	HB	56	0.0714	0.259	0	1
	EPS	41	1.029	2.2	–3.785	5.512
	ROA	34	0.0654	0.271	–0.618	0.484
**(6) Commercial banks**						
	SR	261	0.008	0.352	–0.8315597	3.96291
	HB	280	0.285	0.452	0	1
	EPS	242	5.92	7.055	–19.023	24.4709
	ROA	236	0.009	0.012	-0.054	0.037
**(7) Engineering**						
	SR	228	0.04	0.268	–0.841	1.295
	HB	228	0.105	0.308	0	1
	EPS	186	10.713	3.743	–65.4	54.84
	ROA	184	0.032	0.115	–0.564	0.652
**(8) Fertilisers**						
	SR	95	0.02	0.168	–0.622	0.376
	HB	95	0.41	0.211	0	1
	EPS	67	11.337	16.47	–10.41	122.29
	ROA	66	2.384	5.061	–0.038	26.8
**(9) Food and personal care products**						
	SR	322	0.061	0.328	–1.02	4.177
	HB	323	0.263	0.441	0	1
	EPS	269	35.713	72.026	–16.9	475.54
	ROA	263	0.091	0.195	–1.961	1.228
**(10) Glass and ceramics**						
	SR	152	0.027	0.242	–0.751	0.991
	HB	152	0.158	0.366	0	1
	EPS	127	3.377	4.484	–7.02	14.3
	ROA	126	0.038	0.122	–0.284	0.48
**(11) Insurance**						
	SR	358	–0.001	0.245	–1.38	1.01
	HB	364	0.143	0.35	0	1
	EPS	261	1.643	2.763	0	17.5
	ROA	235	0.043	0.11	–0.813	0.46
**(12) Investment banks**						
	SR	267	–0.062	0.358	–1.974	1.007
	HB	294	0.071	0.258	0	1
	EPS	212	1.491	9.801	–28.594	77.478
	ROA	212	–0.038	0.403	–3.232	2.119
**(13) Leasing companies**						
	SR	98	–0.12	0.538	–3.248	1.007
	HB	98	0.214	0.412	0	1
	EPS	75	0.686	4.295	–18.196	10.249
	ROA	74	–0.004	0.102	–0.459	0.375
**(14) Leather and tanneries**						
	SR	76	0.05	0.26	–0.634	0.972
	HB	76	0.526	0.503	0	1
	EPS	65	28.744	56.109	–56.04	201.65
	ROA	64	0.022	0.415	–2.649	1.751
**(15) Miscellaneous**						
	SR	304	0.034	0.317	–0.801	3.354
	HB	304	0.105	0.307	0	1
	EPS	250	1.814	9.414	–70.42	45.8
	ROA	245	0.002	0.144	–1.082	0.534
**(16) Modarabas**						
	SR	301	–0.014	0.251	–2.079	0.646
	HB	308	0.071	0.258	0	1
	EPS	275	1.008	2.179	–6.441	14.006
	ROA	268	0.031	0.089	–0.522	0.521
**(17) Oil and gas extrapolation**						
	SR	76	0.04	0.198	–0.591	0.463
	HB	76	0.368	0.486	0	1
	EPS	65	25.796	16.716	4.4	82.87
	ROA	66	0.196	0.103	0.023	0.487
**(18) Oil and gas distributions**						
	SR	114	0.013	0.198	–0.699	0.522
	HB	114	0.263	0.442	0	1
	EPS	92	22.953	26.558	–39.05	93.76
	ROA	90	0.075	0.062	–0.046	0.212
**(19) Pharmaceuticals**						
	SR	155	0.052	0.249	–0.958	0.884
	HB	156	0.263	0.442	0	1
	EPS	156	19.782	40.327	–74.3	230.56
	ROA	153	0.123	0.094	–0.126	0.394
**(20) Power generations and gas distributions**						
	SR	221	0.008	0.217	–0.784	0.826
	HB	247	0.053	0.224	0	1
	EPS	197	1.611	6.301	–33.9	14.3
	ROA	191	0	0.278	–3.173	0.324
**(21) Synthetic and rayon**						
	SR	187	0.016	0.206	–0.9	0.928
	HB	190	0.105	0.308	0	1
	EPS	175	–1.714	29.858	–352.93	22.1
	ROA	170	0.036	0.177	–0.529	1.7
**(22) Refinery**						
	SR	54	0.036	0.272	–0.77	0.552
	HB	57	0.474	0.504	0	1
	EPS	47	25.344	28.25	–6.07	100.61
	ROA	47	0.047	0.062	–0.112	0.147
**(23) Sugar and allied industries**						
	SR	542	0.038	0.458	–4.939	5.33
	HB	551	0.158	0.365	0	1
	EPS	486	1.841	10.711	–51.35	47.6
	ROA	480	0.012	0.137	–1.207	0.784
**(24) Technology and communication**						
	SR	115	–0.017	0.301	–0.889	0.758
	HB	152	0.158	0.366	0	1
	EPS	111	0.471	4.688	–14.77	14.76
	ROA	107	0.018	0.211	–1.507	0.607
**(25) Textile composite**						
	SR	879	0.02	0.241	–1.044	1.302
	HB	893	0.263	0.441	0	1
	EPS	809	6.751	27.802	–265.7	287.73
	ROA	793	–0.325	9.827	–276.46	7.675
**(26) Textile spinning**						
	SR	1,457	0.014	0.267	–1.522	1.346
	HB	1,482	0.158	0.365	0	1
	EPS	1,342	3.012	43.087	–489.38	846.76
	ROA	1,319	0.001	0.129	–1.232	1.082
**(27) Textile weaving**						
	SR	202	–0.015	0.466	–3.175	1.681
	HB	209	0.211	0.409	0	1
	EPS	177	0.057	10.227	–106.4	48.11
	ROA	173	25.309	254.415	–4	3211.73
**(28) Tobacco**						
**Tob.**						
	SR	57	0.113	0.312	–1.029	0.865
	HB	57	0.579	0.498	0	1
	EPS	51	18.289	34.919	–24.07	158.04
	ROA	50	0.149	0.182	–0.151	1.024
**(29) Transport**						
**Trans.**						
	SR	53	0.052	0.275	–0.441	0.864
	HB	57	0.105	0.31	0	1
	EPS	50	3.336	10.65	–16.75	25.63
	ROA	47	0.041	0.165	–0.258	0.592
**(30) Vanaspati and allied industries**						
**V&AI**						
	SR	76	–0.018	1.352	–9.289	5.961
	HB	76	0.368	0.486	0	1
	EPS	61	0.787	14.068	–51.1	39.7
	ROA	61	–0.277	0.621	–2.746	0.463

Checking of stationarity of variables is very important before running the required tests on the panel data. Referring Table 3, researchers have used the Fisher type augmented dickey fuller (ADF) test to check the stationarity of study variables in all PSX sectors. The alternate hypothesis considers the absence of unit root or stationarity of the panel, whereas the null hypothesis considers unit root or non-stationarity of the panel data. The results reveal that null hypotheses of unit roots for few variables were rejected, which indicates the stationarity of these variables. In contrast, non-stationarity problem was observed in few other variables, which was removed by taking the first difference. According to the stationarity results, stock returns and herd behaviour were not having stationarity problem at their levels in almost all sectors, i.e., integrated of order I (0). Whereas the macroeconomic variables, i.e., INR, IR, ER, and fundamental variables, i.e., ROA and EPS found stationarity after taking the first difference. Hence, it is finally concluded that all these variables are integrated at the order I (1). The use of the ARDL model has been led by this heterogeneous stationarity of various variables.

**TABLE 3:** Stationarity results.

S#	Sectors	Stationarity Levels	ER	IR	INR	
	All Sectors	At level	14.512	12.267	28.71	
			–0.934	–0.976	–0.231	
		1st Difference	75.381	36.826	275.488	
			(0.000)***	(0.045)**	(0.000)***	

	**Sectors**	**Stationarity Levels**	**SR**	**HB**	**EPS**	**ROA**

1	AA	At level	32.165	57.676	53.47	66.657
			(0.003)***	(0.000)***	(0.000)***	(0.000)***
2	APA	At level	221.918	66.83	18.488	39.801
			(0.000) ***	(0.000) ***	–0.423	(0.002) ***
		1st-Difference			112.981	
					(0.000) ***	
3	C&EG	At level	112.914	57.676	69.47	16.764
			(0.000) ***	(0.000) ***	(0.000) ***	–0.269
		1st-Difference				111.013
						(0.000) ***
4	CEMF	At level	137.559	41.4	0.08	7.847
			(0.000) ***	(0.000) ***	–0.146	–0.448
		1st-Difference			18.468	14.92
					(0.0180)**	(0.060) *
5	Cem.	At level	305.529	3.378	56.832	80.888
			(0.000) ***	–1	(0.040) **	(0.000) ***
		1st-Difference		281.117		
				(0.000) ***		
6	CEMF	At level	137.559		12.115	7.847
			(0.000) ***		–0.1461	–0.448
		1st-Difference			18.468	14.92
					(0.018) **	(0.060) *
7	CB	At level	298.487	370.31	171.75	91.979
			(0.000) ***	(0.000) ***	(0.000) ***	(0.000) ***
8	Eng.	At level	212.672	208.384	184.977	46.759
			(0.000) ***	(0.000) ***	(0.000) ***	(0.036) **
9	Fer.	At level	106.749	92.8566	24.4979	19.5778
			(0.000) ***	(0.000) ***	(0.006) ***	(0.033) **
10	F&PCP	At level	249.911	150.484	104.249	83.526
			(0.000) ***	(0.000) ***	(0.000) ***	(0.000) ***
11	G&C	At level	154.475	164.147	36.9691	36.958
			(0.000) ***	(0.000) ***	(0.002) ***	(0.002) ***
12	Ins.	At level	454.91	158.153	136.352	86.804
			(0.000) ***	(0.000) ***	(0.000) ***	(0.003) ***
13	IB/IC/SC	At level	354.882	217.353	101.697	200.87
			(0.000) ***	(0.000) ***	(0.000) ***	(0.000) ***
14	LC	At level	92.83	120.633	20.8154	39.229
			(0.000) ***	(0.000) ***	(0.010)***	(0.000) ***
15	L&T	At level	82.454	87.451	31.343	35.613
			(0.000) ***	(0.000) ***	(0.000) ***	(0.000) ***
16	Misc.	At level	266.221	475.128	76.139	82.848
			(0.000) ***	(0.000) ***	(0.000) ***	(0.000) ***
17	Mod.	At level	327.703	227.703	202.053	309.9
			(0.000) ***	(0.000) ***	(0.000) ***	(0.000) ***
18	O&GD	At level	146.832	149.21	38.786	8.544
			(0.000) ***	(0.000) ***	(0.000) ***	–0.7412
		1st-Difference				85.67
						(0.000) ***
19	O&GE	At level	89.43	32.8502	11.232	13.331
			(0.000) ***	(0.000) ***	–0.188	–0.1
		1st-Difference			36.277	59.434
					(0.000) ***	(0.000) ***
20	Pharma.	At level	116.106	223.815	23.68	30.368
			(0.000) ***	(0.000) ***	–0.165	(0.034) **
		1st-Difference			99.135	
					(0.000) ***	
21	PG&GD	At level	261.186	194.791	113.838	88.018
			(0.000) ***	(0.000) ***	(0.000) ***	(0.000) ***
22	S&R	At level	179.316	179.317	58.172	37.034
			(0.000) ***	(0.000) ***	(0.000) ***	(0.011) ***
23	Ref.	At level	110.96	38.8329	14.993	19.304
			(0.000) ***	(0.000) ***	(0.020) ***	(0.003) ***
24	S&AI	At level	517.97	312.047	163.962	249.538
			(0.000) ***	(0.000) ***	(0.000) ***	(0.000) ***
25	T&C	At level	144.609	164.147	35.542	27.087
			(0.000) ***	(0.000) ***	(0.000) ***	(0.000) ***
26	TC	At level	803.41	785.344	401.577	384.665
			(0.000) ***	(0.000) ***	(0.000) ***	(0.000) ***
27	TW	At level	217.923	60.029	58.679	94.364
			(0.000) ***	(0.000) ***	(0.000) ***	(0.000) ***
28	Trans.	At level	78.283	17.874	1.451	9.994
			(0.000) ***	(0.006) **	–0.962	–0.124
		1st-Difference			43.991	42.053
					(0.000) ***	(0.000) ***
29	V&AP	At level	107.985	45.275	20.45	87.423
			(0.000) ***	(0.000) ***	(0.008) ***	(0.000) ***

*Statistical significance at 10% level; **Statistical significance at 5% level; ***Statistical significance at 1% level.

Pooled mean group 0 results have been shown in Table 4 for various sectors of PSX. The table indicates short-term and also long-term results. The PMG method has been applied to the herding results of the CSAD model. Furthermore, herding was not found in PSX; hence, PSX itself was not a part of this analysis. Only those sectors that show herding were considered suitable for applying this analysis. The error correction coefficient is required to be significant and also negative as per the PMG model. The error correction depicts the speed of adjustment, whereas the negative coefficient indicates a causal relationship between dependent and independent variables by exhibiting a degree of convergence and correction from short term to long term. A negative sign of the coefficient has confirmed the presence of a long-term relationship. All selected PSX sectors fulfil the criteria of the PMG model because of the negative coefficient of error correction. Differences in different sectors can be observed for the significance of variables in the long run and the short run. In the long run, it can be observed that herding has a significant and negative relationship with the stock returns of the majority of the sector except for textile weaving, textile composite, sugar and allied refinery, miscellaneous, leather and tanneries, leasing companies, investment banks, and food and personal care products.

**TABLE 4:** Regression results (PMG technique).

S #	Sectors	Time Span	SR	ECM	ER	IR	INR	HB	EPS	ROA
1	AA	Long Run			0.003 (00.000)***	-0.043 (0.000)***	–0.004 (00.440)	–12.705 (0.000)***	0.002 (0.006)***	
		Short Run	0.369 (0.000)***	–1.042 (0.000)***	–0.002 (0.240)	–0.003 (0.768)	0.0175 (00.000)***	13.020 (0.000)***	0.055 (0.006)***	
2	APA	Long Run			0.005 (0.000)***	–0.0372 (0.000)***	–0.0119 (0.000)***	–00.214 (0.000)***	0.002 (0.036)**	
		Short Run	0.223 (0.000)***	–1.193 (0.000)***	0.005 (0.064)*	0.042 (0.101)	0.026 (0.001)***	0.124 (0.026)**	0.0129 (0.010)	
3	C&EG	Long Run			0.003 (0.000)***	–0.069 (0.000)***	0.0167 (0.040)**	–0.553 (0.000)***		0.911 (0.000)***
		Short Run	0.455 (0.000)***	–1.125 (0.000)***	–00.001 (0.757)	0.060 (0.033)**	–0.005 (0.264)	0.247 (0.019)**		10.108 (0.163)
4	Cem.	Long Run			0.007 (0.000)***	–0.034 (0.000)***	–0.032 (0.000)***	–0.416 (0.000)***		0.047 (0.026)**
		Short Run	0.108 (0.000)***	–1.155 (0.000)***	0.008 (0.000)***	0.071 (0.000)***	0.031 (0.000)***	0.080 (0.011)**		0.324 (0.009)***
5	CEMF	Long Run			0.002 (0.001)***	–0.024 (0.310)	0.024 (0.130)	–0.286 (0.003)***	0.008 (0.042)**	
		Short Run	–0.174 (0.000)***	–1.199 (0.000)***	–00.002 (0.241)	–0.040 (–0.280)	–0.008 (0.463)	0.005 (0.965)	0.178 (0.447)	
6	CB	Long Run			–00.001 (0.049)**	0.051 (0.000)***	–0.066 (0.000)***	–0.143 (0.000)***	0.001 (0.850)	
		Short Run	0.268 (0.000)***	–1.339 (0.000)***	0.007 (0.000)***	0.034 (0.015)**	0.064 (0.000)***	–0.058 (0.025)**	0.081 (0.000)***	
7	Eng.	Long Run			0.001 (0.844)	–0.032 (0.000)***	–0.003 (0.541)	–0.209 (0.001)***	0.004 (0.053)*	
		Short Run	0.515 (0.000)***	–1.205 (0.000)***	0.003 (0.004)***	–0.002 (0.869)	0.006 (0.200)	0.184 (0.000)***	0.004 (0.838)	
8	Fer.	Long Run			0.001 (0.018)**	0.003 (0.658)	–0.003 (0.441)	–0.057 (0.177)	0.294 (0.000)***	
		Short Run	–00.089 (0.000)***	–1.151 (0.000)***	–00.005 (0.012)**	–0.004 (0.457)	0.016 (0.000)***	0.002 (0.903)	0.457 (0.000)***	
9	FPCP	Long Run			0.007 (0.105)	0.005 (0.543)	–0.007 (0.169)	0.151 (0.000)***		0.016 (0.000)***
		Short Run	–00.077 (0.000)***	–1.025 (0.000)***	0.006 (0.004)***	–0.007 (0.257)	0.029 (0.018)**	–0.046 (0.002)***		0.403 (0.107)
10	G&C	Long Run			0.001 (0.129)	–0.046 (0.000)***	0.001 (0.856)	–0.016 (0.825)	0.008 (0.045)**	
		Short Run	0.417 (0.000)***	–00.964 (0.000)***	0.005 (0.000)***	0.011 (0.241)	–0.004 (0.230)	–0.007 (0.898)	0.014 (0.096)*	
11	Ins.	Long Run			–00.002 (0.000)***	–0.013 (0.337)	–0.027 (0.000)***	–0.449 (0.000)***		0.087 (0.000)***
		Short Run	0.602 (0.000)***	–00.920 (0.000)***	0.005 (0.000)***	0.002 (0.847)	0.013 (0.070)*	0.191 (0.000)***		0.0985 (0.032)***
12	IB/IC/SC	Long Run			–00.002 (0.003)***	–0.055 (0.039)**	0.003 (0.817)	0.217 (0.000)***	0.021 (0.000)***	
		Short Run	0.714 (0.000)***	–1.011 (0.000)***	0.004 (0.005)***	–0.011 (0.491)	–0.009 (0.111)	–0.326 (0.000)***	0.135 (0.098)*	
13	LC	Long Run			–00.007 (0.149)	–0.009 (0.715)	–0.013 (0.364)	0.103 (0.060)*	0.931 (0.010)**	
		Short Run	0.222 (0.069)*	–1.084 (0.001)**	0.004 (0.075)*	–0.010 (0.470)	0.002 (0.804)	–0.035 (0.431)	0.056 (0.023)**	
14	L&T	Long Run			0.075 (0.407)	–0.073 (0.000)***	0.029 (0.000)***	0.986 (0.000)***	0.006 (0.000)***	
		Short Run	0.093 (0.118)	–1.167 (0.058)*	0.004 (0.014)**	0.080 (0.232)	0.027 (0.061)*	–0.673 (0.018)**	0.002 (0.696)	
15	Misc.	Long Run			0.001 (0.007)***	–0.026 (0.003)***	–0.005 (0.279)	–0.202 (0.003)***	0.066 (0.005)***	
		Short Run	0.308 (0.000)***	–1.112 (0.000)***	0.005 (0.002)***	0.020 (0.203)	0.008 (0.111)	0.147 (0.001)***	0.031 (0.020)**	
16	Mod.	Long Run			0.002 (0.000)***	–0.228 (0.000)***	0.145 (0.000)***	–1.344 (0.000)***		0.077 (0.000)***
		Short Run	1.093 (0.000)***	–00.983 (0.000)***	–0.006 (0.239)	–0.063 (0.000)***	–0.106 (0.000)***	0.329 (0.000)***		0.067 (0.000)***
17	O&GE	Long Run			–0.010 (0.000)***	0.078 (0.001)***	–0.056 (0.000)***	–0.233 (0.000)***		1.926 (0.000)***
		Short Run	1.357 (0.008)***	–1.286 (0.006)***	0.008 (0.031)**	–0.026 (0.493)	0.056 (0.000)***	0.240 (0.001)***		–0.635 (0.402)
18	O&GD	Long Run			0.003 (0.000)***	0.005 (0.267)	–0.004 (0.444)	–0.242 (0.000)***		1.066 (0.010)**
		Short Run	–00.409 (0.000)***	–1.375 (0.000)***	–0.001 (0.539)	0.009 (0.681)	0.0357 (0.009)***	0.088 (0.010)**		2.834 (0.011)**
19	Pharma.	Long Run			0.004 (0.000)***	–0.017 (0.081)*	–0.017 (0.003)***	–0.277 (0.000)***	0.006 (0.668)	
		Short Run	0.107 (0.000)***	–1.079 (0.000)***	0.008 (0.000)***	0.077 (0.001)***	0.008 (0.068)*	0.124 (0.000)***	0.006 (0.077)*	
20	PG&D	Long Run			0.001 (0.011)**	–0.034 (0.000)***	0.0175 (0.002)***	–0.106 (0.191)	0.008 (0.016)**	
		Short Run	0.181 (0.000)***	–1.181 (0.000)***	–0.001 (0.930)	–0.018 (0.000)***	–0.021 (0.004)***	0.236 (0.019)**	0.040 (0.006)***	
21	S&R	Long Run			0.001 (0.053)**	–0.011 (0.276)	–0.008 (0.243)	0.118 (0.090)**	0.005 (0.265)	
		Short Run	0.066 (0.000)***	–00.917 (0.000)***	0.004 (0.001)***	0.005 (0.618)	0.006 (0.217)	–0.152 (0.003)***	0.021 (0.048)**	
22	Ref.	Long Run			0.004 (0.587)	–0.0117 (0.465)	–0.0144 (0.106)	0.118 (0.057)*		0.089 (0.000)***
		Short Run	0.276 (0.000)***	–1.570 (0.000)***	0.007 (0.000)***	0.005 (0.892)	0.016 (0.006)***	0.002 (0.744)		0.054 (0.667)
23	S&AI	Long Run			0.001 (0.000)***	–0.048 (0.000)***	0.008 (0.007)***	–0.025 (0.000)***	0.274 (0.041)**	
		Short Run	0.394 (0.000)***	–1.123 (0.000)***	0.007 (0.000)***	0.065 (0.000)***	–0.018 (0.000)***	–0.168 (0.000)***	0.221 (0.626)	
24	T&C	Long Run			0.009 (0.516)	–0.054 (0.055)**	0.016 (0.370)	–0.245 (0.008)***	0.0127 (0.017)**	
		Short Run	0.447 (0.000)***	–1.103 (0.000)***	0.004 (0.250)	–0.005 (0.843)	0.014 (0.330)	0.348 (0.006)***	0.012 (0.592)	
25	TC	Long Run			0.002 (0.000)***	–0.032 (0.000)***	0.001 (0.709)	0.091 (0.001)***	0.064 (0.016)**	
		Short Run	0.158 (0.000)***	–1.088 (0.000)***	0.003 (0.000)***	0.031 (0.000)***	0.006 (0.112)	–0.022 (0.096)*	0.066 (0.045)**	
26	TW	Long Run			0.005 (0.000)***	0.001 (0.934)	–0.014 (0.202)	0.272 (0.001)***		0.067 (0.000)***
		Short Run	–00.503 (0.000)***	–1.157 (0.000)***	0.003 (0.009)***	–0.036 (0.229)	0.030 (0.001)***	–0.130 (0.275)		0.046 (0.843)
27	Trans.	Long Run			0.004 (0.042)**	–0.151 (0.000)***	0.036 (0.014)**	–0.676 (0.001)***	0.033 (0.097)*	
		Short Run	1.282 (0.000)***	–1.030 (0.000)***	0.006 (0.428)	0.036 (0.535)	–0.038 (0.021)**	0.192 (0.007)***	0.329 (0.375)	
28	V&AI	Long Run			0.004 (0.877)	–0.005 (0.894)	–0.001 (0.930)	–0.048 (0.746)	0.005 (0.000)***	
		Short Run	0.180 (0.022)**	–1.023 (0.000)***	–0.007 (0.129)	–0.175 (0.329)	0.024 (0.452)	0.126 (0.376)	0.005 (0.620)	

*Statistical significance at 10% level; **Statistical significance at 5% level; ***Statistical significance at 1% level.

Conversely, in the short run, herding has a positive relationship with all sectors’ stock returns except some sectors, which negatively associates with the stock returns. The possible reasoning for this negative relationship is the price destabilisation caused by herding. Furthermore, herding negatively predicts long-term returns and positively predicts short-term returns. However, some sectors have shown an insignificant relationship between stock returns and herd behaviour. Furthermore, in few industries, short-run results are insignificant, and long-run effects are significant, i.e., textile weaving, refinery, leasing companies, and close-end mutual funds. Conversely, the results of gas development and the power generation sector demonstrate long-run insignificant and short-run significant results.

As far as the long-run exchange rate is concerned, a significant positive relationship was found between stock returns and exchange rate in various sectors of PSX except for few financial sectors (oil and gas exploration, leasing companies, investment banks, insurance companies, and commercial banks). An inverse relationship was observed in these sectors between stock returns and exchange rate. However, in the short run, most of the sectors showed a positive relationship except fertilisers. The frequency of this relationship was observed to be similar across all sectors.

Inflation rate and interest rate have an inverse relationship with stock returns. As far as the long run is concerned, most sectors indicated inverse connection between stock returns and interest rates except oil and gas exploration and commercial banks, where these two sectors showed a positive relationship. On the other hand, in the short run, a positive relationship was found in few sectors (sugar and allied industries, pharmaceuticals, commercial banks, cement, cable and electrical goods, and automobile parts and accessories), and few other sectors also showed a negative relationship. However, in the short run, most of the sectors showed an insignificant connection. Considering the inflation rate, most sectors indicated a negative relationship between stock returns and interest rates, but some sectors also showed a positive relationship, i.e., transport, sugar, and allied industries, power generation and gas distribution, modarabas, and cable and electrical goods. Furthermore, insignificant results were found for the short run (interest rate) and long run (inflation rate) for these mentioned sectors. Other than the long run, a positive relationship was found in the short run in many sectors. However, an insignificant relationship was also found in few sectors.

As far as the results of fundamental factors are concerned, for every sector, either the results of ROA or EPS are shown at one time based on their significance in the short run or long run or at both times. Various sectors showed a significant relationship between stock returns and EPS, and few sectors showed significant values for ROA in the short run and long run, as reported in the table. Positive ROA and EPS provide strength to the stock returns. The enhanced level of earnings can give a positive signal to the investors while boosting their investment behaviour. High return on assets allows a corporation to achieve good returns on their investments by utilising raising profits in the stock market and by providing an opportunity to investors for purchasing more stocks of the corporation.

## Discussion

The mixed evidence has been observed for the association between herding and stock returns, and the results of current research have been fully supported by previous researchers. The academicians have found the positive and negative impact of herding on stock returns (Jeon and Moffett, 2010; Celiker et al., 2015). The stock returns assume negative effect of herding due to price destabilisation (Sias and Starks, 1997). Moreover, in the short run, investors’ limited information processing capacity leads them to increase their stock investments, thereby generating high returns. Based on artificial spark, the bullish trend can also be observed in the short run. On the other hand, the investors may find sufficient time to process information in the long run, which leads to avoid investments or seems the stock investment as a favourable avenue. Literature also revealed that herd behaviour negatively forecasts long-run returns and positively predicts short-run returns (Dasgupta et al., 2011). The mixed results of current research are also backed by Zheng et al. (2015), who discovered the positive short-run and long-run linkage between stock returns and herd behaviour.

Previous researchers that include the researchers of Pakistan also supported the positive relationship between exchange rate and stock returns (Khan, 2014; Afshan et al., 2018; Khan et al., 2018) and negative relationship (Menike, 2006) in both long run (Mitra, 2017; Türsoy, 2017; Ndlovu et al., 2018) and short run (Bashir et al., 2016), thereby providing support to the results of this study. However, Gokmenoglu et al. (2021) explained that stock market returns are not affected by exchange rates. Some theoretical backgrounds are also available in explaining such a relationship; i.e., portfolio balance theory (1983) describes the negative connection, whereas theory of goods market (1980) presents the positive linkage between exchange rate and stock returns. It has been observed that the exchange rate stability reinforces the stock returns, and fluctuations in exchange rate leave unfavourable effects on stock returns in both long run and short run.

Results of this study are also in line with the previous studies where the positive effect of interest rate on stock returns (Kibria et al., 2014; Ndlovu et al., 2018) and also the negative impact (Khan, 2014; Afshan et al., 2018) has been established. The increased cost of debts negatively influences stock returns; however, increased interests sometimes optimise stock returns. Interest rate and inflation rate have similar effects on stock returns. Inflation rate also influenced the stock returns in both ways, i.e., positive and negative in both long run and short run, and literature supports these findings (Butt et al., 2010; Mahmood et al., 2015; Khan et al., 2018; Ndlovu et al., 2018; Ifeanyi et al., 2021).

The fundamental variables, i.e., return on assets and earnings per share, have dual effects on stock returns for both long and short run, and the findings are backed by previous researchers (Mirfakhr-Al-Dini et al., 2011; Hatta, 2012; Zulkarnaen et al., 2012; Abdulmannan and Faturohman, 2015; Ahmed, 2018; Saragih, 2018). The higher earnings and the good EPS of a firm boost investors to make stock investments, which result in optimised returns. Agrawal and Bansal (2021) explained that EPS positively affect stock returns.

## Practical Implications

The current research has some implications for investors, policymakers, and managers. Minimisation of herd behaviour is possible if information is appropriately provided to the investors. Furthermore, investors should be very much clear about the firm-specific information. Moreover, to make rational and appropriate decision, they must know the herding contents. With respect to stock returns, herding can be useful to generate the returns; therefore, investors should keep in mind that herd behaviour is not something bad or undesirable phenomenon. In this situation, concreate efforts should be made by the investors for taking the thorough information about stock specifications, because when investors are motivated toward herding, they usually make investments in the security in which other investors get higher returns. On the other hand, when herd behaviour causes negative returns, the above-discussed precautions must be followed by the investors to minimise such losses. This study also suggests a couple of guidelines to regulatory authorities as to how to maximise stock returns. The policymakers must thoroughly understand the mechanism with which herding affects the stock returns. They can place an effort in magnifying the stock returns even in the presence of herd behaviour. They can ensure the best possible working of macroeconomic variables in generating higher returns. The efficient performance of inflation rate, interest rate, and exchange rate paves the way for the effective working of the economy and leads investors to consider the stock investment as a favourable avenue. Besides, substantial investment in stock generates positive returns. In such a perspective, managers can try their level best to enhance the functioning of fundamental factors (earnings per share and return on assets). The auspicious earnings create robust signals for the investors by boosting their investment attitude. Besides, preeminent return on assets optimises the stock investment potential of investors.

## Limitations and Future Directions

This study has provided some valuable insights into the current body of knowledge; however, it also has few limitations. These limitations can be exploited by the future researchers to produce more valuable and efficient research. The existing research has relied totally on secondary data to investigate the herd behaviour. Herding can be measured in Pakistan by the usage of primary data. Primary data can be very useful to measure the herd behaviour as it may be based an investor’s reallife experiences, and for this, the future researchers can also devise a herding scale. Moreover, future researchers can investigate herding in the South Asian stock markets, and herding phenomenon in these markets can be compared with each other.

## Conclusion

This study investigates the impact of herd behaviour on stock returns with some macroeconomic and fundamental control variables at the industry level in Pakistan Stock Exchange. Herding has been detected in most of the sectors of PSX, which further positively and negatively influence stock returns. However, findings revealed that some sectors have an insignificant association between herd behaviour and stock returns. The connection between control variables (earnings per share, return on assets, exchange rate, inflation rate, and interest rate) and stock returns is significant in some sectors. On the other hand, some sectors exposed insignificant relationship. These diverse findings have been experienced in both the long run and short run.

## Data Availability Statement

The raw data supporting the conclusions of this article will be made available by the authors, without undue reservation.

## Author Contributions

All authors have contributed equally to complete the research manuscript and approved the submitted version.

## Conflict of Interest

The authors declare that the research was conducted in the absence of any commercial or financial relationships that could be construed as a potential conflict of interest.

## Publisher’s Note

All claims expressed in this article are solely those of the authors and do not necessarily represent those of their affiliated organizations, or those of the publisher, the editors and the reviewers. Any product that may be evaluated in this article, or claim that may be made by its manufacturer, is not guaranteed or endorsed by the publisher.
